# Fumarate hydratase-deficient renal cell carcinoma cells respond to asparagine by activation of the unfolded protein response and stimulation of the hexosamine biosynthetic pathway

**DOI:** 10.1186/s40170-020-00214-9

**Published:** 2020-08-03

**Authors:** Rony Panarsky, Daniel R. Crooks, Andrew N. Lane, Youfeng Yang, Teresa A. Cassel, Teresa W.-M. Fan, W. Marston Linehan, Jeffrey A. Moscow

**Affiliations:** 1 Urologic Oncology Branch, Center for Cancer Research, National Cancer Institute, NCI Shady Grove Room 5 W460, 9609 Medical Center Drive, Bethesda, MD 20892-9739 USA; 2 Center for Environmental and Systems Biochemistry, University of Kentucky, Lexington, KY USA; 3 Markey Cancer Center and Department of Toxicology and Cancer Biology, University of Kentucky, Lexington, KY USA

**Keywords:** Fumarate hydratase, Renal cell carcinoma, Asparagine metabolism, Unfolded protein response, SIRM

## Abstract

**Background:**

The loss-of-function mutation of fumarate hydratase (FH) is a driver of hereditary leiomyomatosis and renal cell carcinoma (HLRCC). Fumarate accumulation results in activation of stress-related mechanisms leading to upregulation of cell survival-related genes. To better understand how cells compensate for the loss of FH in HLRCC, we determined the amino acid nutrient requirements of the FH-deficient UOK262 cell line (UOK262) and its FH-repleted control (UOK262WT).

**Methods:**

We determined growth rates and survival of cell lines in response to amino acid depletion and supplementation. RNAseq was used to determine the transcription changes contingent on Asn and Gln supplementation, which was further followed with stable isotope resolved metabolomics (SIRM) using both [U- ^13^C,^15^N] Gln and Asn.

**Results:**

We found that Asn increased the growth rate of both cell lines in vitro. Gln, but not Asn, increased oxygen consumption rates and glycolytic reserve of both cell lines. Although Asn was taken up by the cells, there was little evidence of Asn-derived label in cellular metabolites, indicating that Asn was not catabolized. However, Asn strongly stimulated Gln labeling of uracil and precursors, uridine phosphates and hexosamine metabolites in the UOK262 cells and to a much lesser extent in the UOK262WT cells, indicating an activation of the hexosamine biosynthetic pathway (HBP) by Asn. Asn in combination with Gln, but not Asn or Gln alone, stimulated expression of genes associated with the endoplasmic reticulum (ER) stress and the unfolded protein response (UPR) in UOK262 to a greater extent than in FH-restored cells. The changes in expression of these genes were confirmed by RT-PCR, and the stimulation of the UPR was confirmed orthogonally by demonstration of an increase in spliced XBP1 (sXBP1) in UOK262 cells under these conditions. Asn exposure also increased both the RNA and protein expression of the HBP regulator GFPT2, which is a transcriptional target of sXBP1.

**Conclusions:**

Asn in the presence of Gln induces an ER stress response in FH-deficient UOK262 cells and stimulates increased synthesis of UDP-acetyl glycans indicative of HBP activity. These data demonstrate a novel effect of asparagine on cellular metabolism in FH-deficient cells that could be exploited therapeutically.

## Background

Kidney cancer is one of the ten most common cancers, and renal cell carcinoma (RCC) composes the vast majority of kidney cancer cases. While more than 90% of all RCC have sporadic etiology, approximately 3-5% are attributed to an underlying germline abnormality. Hereditary leiomyomatosis and renal cell carcinoma (HLRCC) is an aggressive form of RCC characterized by germline inactivating mutation of fumarate hydratase (FH), followed by somatic loss of the remaining wild-type allele. FH is a key metabolic enzyme of the mitochondrial tricarboxylic acid (TCA) or Krebs cycle, and as a result of FH loss-of-function, the metabolite fumarate accumulates. This lesion has two major oncogenic effects: it directly stimulates a metabolic transition to Warburg metabolism, through the disruption of the Krebs cycle, leading the cells to shift to alternative pathways in order to sustain a balance of energy required for survival and proliferation; and the excess fumarate results in accumulation of members of the hypoxia inducible factor family of transcription factors [1, 2]. Thus, FH acts practically as a tumor suppressor gene.

The loss of FH gene function in HLRCC would seem to provide a unique metabolic vulnerability that could be exploited for therapeutic benefit. This cell line has been shown to have diminished oxidative phosphorylation capacity, increased lactate production, and rapid glycolytic flux, all indicating impairment of Krebs cycle activity and characteristic of the Warburg effect [3]. Metabolic studies of the parental UOK262 cell line (also referred to as UOK262KO) compared to UOK262 cells transfected with an FH expression vector (UOK262WT) using isotopically labeled glucose and glutamine (Gln) showed that FH deficiency resulted in increased glucose utilization via glycolysis with increased production of glucose-derived lactate, increased use of the oxidative branch of the pentose-phosphate pathway (PPP) to generate both ribose and NADPH and that Gln is used preferentially to generate α-ketoglutarate [4]. These studies also indicated the presence of high levels of dihydroorotate dehydrogenase activity needed to provide pyrimidine nucleotide pools for RNA synthesis [2].

Excess fumarate itself also acts as an oncometabolite. The oncogenic pathways of renal cell carcinomas converge upon the dysregulation of the hypoxia inducible factor (HIF) family of transcription factors. For example, mutations of the oncogenic driver Von Hippel Landau (VHL) gene impair the ability of the VHL protein to ubiquitinate HIFs for their further degradation. The accumulation of fumarate in FH−/− cells inhibits the Hif1α prolyl hydroxylase, resulting in failure to be recognized by VHL and thus accumulation of HIFs [5].

Multiple cancer cell lines are highly dependent on continuous supply of Gln for sustaining their energy balance and the demand for growing biomass. Gln is the most abundant amino acid in the human plasma that allows cancer cells to adapt the metabolism for maximal utilization of Gln along with glucose. Gln fuels the tricarboxylic acid (TCA) cycle through anaplerosis and contributes to the synthesis of lipids, nucleotides, and non-essential amino acids. Some cells, however, develop Gln independence by switching to asparagine (Asn) as an alternative source of cancer metabolism. Asn may be supplied exogenously from the extracellular environment or may be synthesized de novo from Gln by upregulating intracellular levels of asparagine synthase (ASNS). Recently, studies have shown that Asn may play a key role as an exchange factor for other amino acids in proliferating cancer cells [6], where intracellular Asn acts as a driver for serine uptake and others amino acids to promote cell growth through activation of mTORC1 pathway. Knocking down the ASNS gene in sarcoma cells combined with depletion of extracellular Asn was demonstrated to attenuate tumor growth in vivo [7]. Finally, inhibition of GCN2, an important element of cellular response to amino acids limitation, sensitized acute lymphoblastic leukemia cells to asparaginase through downregulation of ASNS [8].

We began our approach to better understand how the UOK262 cell line adapts to FH deficiency by identifying the nutrient requirements for cell growth in vitro. Our studies quickly focused on Asn as a nutrient required for optimal in vitro growth in Gln-supplemented medium. By comparing FH-deficient cells to the same cell line transfected with the FH gene, mRNA-seq studies demonstrated that Asn stimulated endoplasmic reticulum (ER) stress and the unfolded protein response (UPR) in the FH-deficient cell line. Metabolic studies showed that the FH-deficient cell line increased the synthesis of uridine nucleotides and hexosamine pathway metabolites in the presence of Asn. These studies showed an unexpected role of Asn to stimulate the UPR in the context of FH-deficiency, and the unexpected role of pyrimidine and hexosamine metabolism in response to the UPR in this model system.

## Methods

### Cell lines

The UOK262 FH^−/−^ (UOK262 also referred to in figures as UOK262KO) cell line was derived from a metastatic site, and the UOK268 FH^−/−^ (UOK268) was derived from a primary tumor of separate patients with the HLRCC syndrome at the Urologic Oncology Branch (UOB) as described previously [2, 3]. The UOK262WT and UOK268WT cell lines were created by the stable reintroduction of the wild-type *FH* gene back into the established cell lines [9, 10]. The cells were maintained at DMEM with 10% FBS, 25 mM glucose, 4 mM Gln, 1 mM sodium pyruvate, 100 units/mL penicillin, and 100 μg/mL streptomycin and split every 2–3 days before reaching confluency. A non-essential amino acid (NEAA) supplement (Sigma-Aldrich, St. Louis, MO, USA) was added to the medium at a dilution of 1:100 unless otherwise noted.

### Cell culture and treatment

For cytotoxicity experiments with various compounds, the cells seeded at 96-well plates (3000/well) in medium (DMEM with 10% FBS, 25 mM glucose, 100 units/mL penicillin, and 100 μg/ml streptomycin) 24 h prior to start of experiments. On the day of the experiment, the appropriate drug dilutions were made using minimal media with addition of Asn, Gln, or both and added on top of the cells. The cell growth assessment was performed after 96 h incubation by means of the sulforhodamine B (SRB) assay. For Western blot and qRT-PCR analysis, the cells seeded at 6-well plates in doubles (350,000/well) 24 h prior to treatment. After the incubation, the appropriate amino acids added to the cells and plates were incubated for 96 h prior to the termination of experiments and trypsinization. The cells were collected and immediately processed or stored at − 80 °C for Western blot analysis and/or RNA extraction. For the reverse experiment, both cell lines were incubated in medium containing both amino acids for 72 h following removal Asn, Gln, or both amino acids for the next 24 h while the control cells were treated with Gln and Asn combined for 96 h.

### Antibodies and drugs

Anti-CHOP antibody (CS2895), anti-p-eIF2α antibody (CS3398), anti-eIF2α antibody (CS2103), anti-p-JunB antibody (CS3177), anti-p-Grp78 antibody (CS3177), anti-p-4E-BP1 antibody (CS9455), anti-p-mTOR antibody (CS2971), anti- mTOR antibody (CS2972), anti-p-p70 kinase antibody (CS9234), anti-p-S6RP(Ser235/236), antibody (CS4856), anti-p-S6RP (Ser240/244), antibody (CS5364), anti-S6RP antibody (CS2317), anti-Grp94 antibody (CS2104), anti-JunD antibody (CS5000), and anti-β-Actin antibody (CS3700) were purchased from Cell Signaling Technology (Boston, MA, USA); anti-XBP1 antibody (AB) was purchased from Abcam (Cambridge, MA, USA).

Chloroquine disulfate (S6628), thapsigargin (T9030), Fli06 (SML0975), tunicamycin (T7765), 5-aminoorotic acid (191213), teriflunomide (SML0936), and DFMO (D193) were obtained from Sigma-Aldrich (St. Louis, MO, USA) and diluted in DMSO or in water according to the manufacturer recommendation.

### Western blot analysis

After trypsinization, cells were collected and centrifuged at 1500 rpm for 10 min at 4 °C. The cell pellet was lysed in RIPA lysis buffer (Sigma-Aldrich, St. Louis, MO, USA) with 1% of phosphatase and protease inhibitor (Cell Signaling Technology, Boston, MA, USA). The homogenate was centrifuged at 14,000 rpm for 5 min 4 °C and the supernatant was collected. The supernatant was stored at − 80 °C for later use. A standard BCA (Sigma-Aldrich, St. Louis, MO, USA) method was used to determine protein concentration. Then, a total of 15 μg protein each lane was subjected to SDS-PAGE gradient gel (4-20%) and transferred to a nitrocellulose membrane following by primary antibody overnight incubation. After 3 washes with TBST buffer, the membranes exposed to secondary antibodies IRDye680 and IRDye800 (LI-COR Biosciences, Lincoln, NE, USA) and subjected to imaging using Odyssey Imaging System (LI-COR Biosciences, Lincoln, NE, USA). The optical density was analyzed using the Image J software (Rawak Software, Inc., Stuttgart, Germany).

### Measurements of OXPHOS and lactic fermentation

The XF96 Extracellular Flux Analyzer (Seahorse Bioscience, North Billerica, MA) was used to detect rapid, real-time changes in cellular respiration and glycolysis rate. The cell lines were cultured in custom XF96 microplates as recommended by the manufacturer [11]. The standard cell seeding density was 25,000/well, and the cells were seeded 24 h prior to the experiment. Immediately following the addition of fresh medium, basal levels of OCR and proton production were first quantified over approximately 20–25 min. Analysis of ECAR reflects lactate excretion and serves as an indirect measure of the glycolysis rate, whereas OCR reflects cellular respiration and is directly determined. Experiments were performed by simultaneously measuring three to five replicates of each cell line. Relative effects were expressed as areas under the curve measurements that were generated by the manufacturer’s software and used to compare the various cell lines, and the observed rates were reported in pMol/min/cell for OCR and mpH/min/cell for ECAR. The total mass of cells at each well was determined using the SRB assay for normalization at the end of the experiment.

### RNA extraction and purification

At the end of treatment with corresponding amino acids, the cells were harvested with trypsin 0.25% and centrifuged at 1500 rpm for 10 min 4 °C. The cell pellet was transferred to − 80 °C for further applications or used immediately for total RNA extraction using the PureLink RNA mini kit (Thermo Fisher Scientific) protocol. The total RNA concentrations were determined using a NanoDrop instrument. The total RNA was stored at − 80 °C for downstream applications.

### Messenger RNA sequencing

Twenty-four mRNA-seq libraries were created and sequenced on a single Hiseq3000 flowcell lane using TruSeq V2 chemistry, 150 bp paired-end. Sequencing quality and yields for the samples were good, with yields ranging between 26 and 41 million reads and percentage of Q30 bases above 92% for all samples. Sample reads were trimmed of adapters and low-quality bases using the Trimmomatic software and then aligned to the human (hg19) reference genome and Ensembl_v70 transcripts using the Star software. The percentage of trimmed reads that aligned to the reference was high, above 94% for all samples. Uniquely mapped read percentages were also high, above 88% for all samples. RNA mapping statistics were calculated using the Picard software. The percentage of mRNA mapped sample bases was high, above 92% for all samples, with coding bases consisting of between 60 and 63% of those bases. Intronic and intergenic base percentages were within expected ranges for mRNA, below 4% in all cases. The percentage of ribosomal bases was good, less than 2% for all samples. Library complexity (i.e., the percentage of non-duplicated fragments) was measured by the number of unique fragments among the mapped reads using Picard’s Markduplicate utility. Sample library complexity was above average, with the percentage of unique fragments between 64 and 72%.

### Quantitative real-time-PCR validating mRNA transcriptome analyses

Gene expression alterations were analyzed by qRT-PCR for upregulated genes in UOK262 (FH null) and UOK262WT (FH restored), and UOK268 (FH null) and UOK268WT (FH restored) cells under various treatments as compared to the untreated control. One microgram of total RNA was reverse-transcribed to cDNA using SuperScript IV reverse transcriptase (Invitrogen) and random hexamers in a final volume of 20 μL. The resulting cDNA volume was diluted ten times and 4 μL of the solution was used for PCR amplification in an ABI 7000 real-time PCR system (Applied Biosystems) as recommended by the manufacturer. Primers and fluorogenic probes were designed by Applied Biosystems in the form of TaqMan Gene Expression Assays for the *DDIT* (Hs00358796_g1), *HSPA5* (Hs00946086_g1), *JUNB* (Hs00357891_s1), *TGFB* (Hs00998133_m1), *DUSP1*(Hs00610256_g1), *XBP1s* (Hs02856596_m1), *GFPT2* (Hs01049561_m1), *CAD* (Hs00983188_m1), and *GDF15* (Hs00171132_m1), and used for the validation of RNASeq results. Expression levels for these genes in UOK268 and UOK 262 were normalized to a control housekeeping gene, *ACTB* (Hs01060665_g1), samples were run in triplicate, and CT values obtained were compared by the Delta CT method. Results are expressed as an average fold-change compared to untreated values from at least three independent experiments.

### Cell culture and ^13^C^15^N-Gln/^13^C^15^N-Asn labeling in vitro

UOK262 FH depleted and replete cell lines were seeded at the 10 cm dishes (1 million cells/well) a day prior the beginning of experiment at base medium (DMEM without pyruvate with 10% FBS, 25 mM glucose, 100 units/mL penicillin, and 100 μg/mL streptomycin). For the Stable Isotope Resolved Metabolomics (SIRM) experiments, the cells were incubated at base medium supplemented with unlabeled Asn, Gln, or both for 72 h. For the final 24 h, the growth medium was changed to fresh medium supplemented with 2 mM [U-^13^C^15^N]-Gln, 100 μM [U-^13^C^15^N]-Asn or combination of labeled and unlabeled amino acids in a CO_2_ incubator maintained at 37 °C. At the end of incubation (96 h), cells were washed with ice-cold PBS three times followed by extraction with acetonitrile/water/chloroform (V/V 2:1.5:1) and separated into polar, lipid, and protein fractions as described elsewhere [12].

### Polar metabolite analysis by NMR spectroscopy

NMR spectra were recorded at 14.1 T on an Agilent DD2 spectrometer with automation in 1.7-mm tubes in a 3-mm inverse HCN cold probe at 15 °C with an acquisition time of 2 s and a presaturation delay of 4 s for ^1^H experiments, using a weak transmitter rf field for saturating the strong solvent signal. 1D ^1^H{^13^C}-HSQC spectra were recorded with an acquisition time of 0.2 s with adiabatic decoupling and a relaxation delay of 1.8 s. Samples were maintained at 6 °C prior to NMR analysis. Compounds were identified from chemical shifts and splitting patterns in 1D, cross-referenced to 2D spectra, using our in-house database [13] and those of HMDB [14]. We used a targeted analysis for a small number of abundant compounds as per protocol for non-SIRM experiments. Whenever possible, we also estimated the ^13^C enrichment at individual positions such as lactate and Ala methyl groups and glucose H1. Data were analyzed using MNova v 10.0 (Mestrelab Research, Santiago de Compostela, Spain). Free induction decays were linear predicted once and zero filled, prior to fourier transformation, the spectra were baseline corrected with a simple third order polynomial. Concentrations were determined by peak integration with normalization to the DSS resonance at 0 ppm, with corrections for partial saturation as described [13].

### Ion chromatography ultrahigh resolution FTMS

Methanol (HPLC grade, ≥ 99.9%, Sigma-Aldrich) was used as the makeup solvent providing after Ion chromatography to assist electrospray in the mass spectrometer. Nano pure water was obtained from a Milli-Q® Integral Water Purification System (Thermo Scientific). Ion chromatography was carried out using a Dionex ICS5000^+^ system equipped with a dual pump (DP), an eluent generator (EG), an autosampler (AS-AP), and a detector/chromatography module (DC). An IonPac AG11-HC-4 μm guard column (2 × 50 mm) followed by an IonPac AS11-HC-4 μm RFIC&HPIC (2 × 250 mm) analytical column was used with a constant temperature at 35 °C, and the column flow rate was kept at 0.38 mL/min. The eluent was injected into a Thermo Fusion Orbitrap mass spectrometer using full scan negative ion mode with a nominal resolution of 360,000 at m/z = 200 as previously described [15]. Metabolites were identified and quantified using the TraceFinder version 3.3 (Thermo Scientific) software using a mix of 88 standards, and corrected for natural abundance ^13^C contributions [16].

### Statistical analysis

All data are presented as means ± SEM. Graph Pad Prism 7 was used for all statistical analysis. One-way ANOVA for multiple comparisons followed by Holm-Sidak corrected unpaired 2-tailed *t* test were used to determine the differences among all groups. *p* < 0.05 was considered to be a significant difference.

## Results

### Amino acid requirements for proliferation of UOK262 cells

**Fig. 1:**
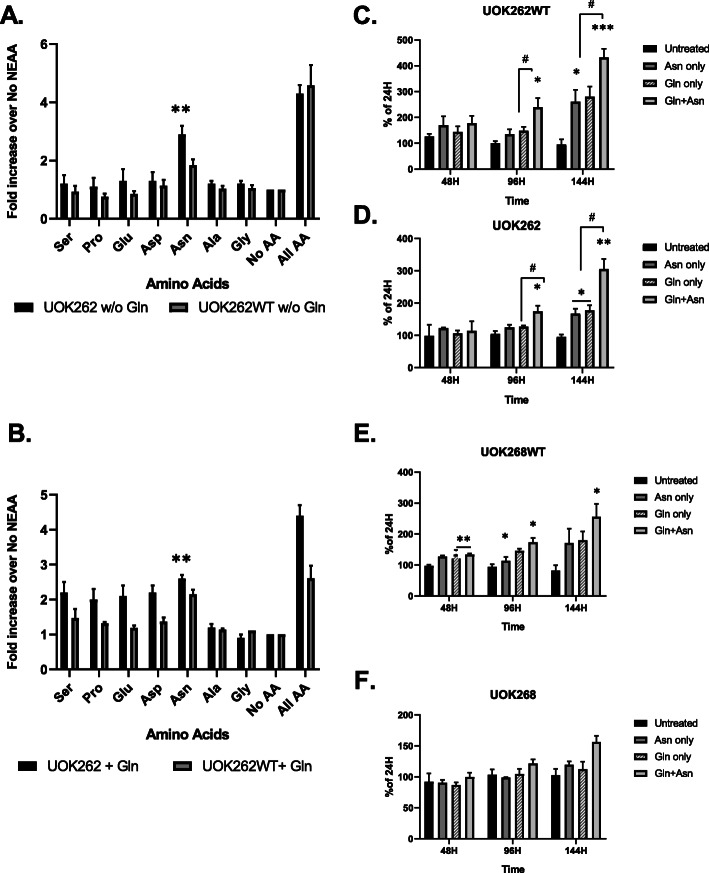
UOK262 and UOK262WT cells growth with NEAA’s in the absence (**a**) or presence (**b**) of 2 mM Gln. The cells were incubated with 100 μM of each component of NEAA separately, together or without any for 48 h. The cell density determined using the SRB assay. UOK262WT (**c**) and UOK262 (**d**) (distinct metastatic site) were subjected to up to 144 h of incubation in the presence of 2 mM Gln, 100 μM Asn, or both amino acids. Cell growth measurements were taken after 48 h, 96 h, and 144 h and expressed as a percentage of cell density at 24 h. UOK268WT (**e**) and UOK268 (**f**) (primary site) cell lines were subjected to similar analysis over time of 144 h to compare to UOK262 lines. Samples were analyzed in triplicate, and the results shown are average cell density with a standard error of the mean. Statistical analysis was performed to compare the untreated versus treated samples using unpaired, two-tailed *t* tests (GraphPad Prism v. 8). (*) *p* < 0.05; (**) *p* < 0.01; (***) *p* < 0.0001. The significance between treatment groups shown as (#) *p* < 0.05

UOK262 cells are routinely grown in DMEM supplemented with non-essential amino acids (NEAA). To identify which of the constituents of this supplement is required for cell growth, we incubated both UOK262 and UOK262WT cell lines in the presence or absence of 2 mM of Gln with each of the seven NEAA for 48 h in high-glucose DMEM medium. Figure 1 shows that robust cell growth was achieved when all seven NEAA were supplemented, whereas in the absence of Gln only Asn supported significant cell growth (Fig. 1a), whereas in the presence of Gln, all of the non-essential amino acids except alanine and glycine supported growth (Fig. 1b). Growth curves over a longer duration revealed that both Gln and Asn significantly contribute to cell growth for both cell lines, but only after the first 96 h of incubation (Fig. 1c and d) indicating that a period of adaptation was required for Asn availability to show an effect on cell growth. A similar pattern was observed with UOK268 FH null and FH-restored (UOK268WT) cell lines (Fig. 1e and f). These results suggest the important role Gln and Asn play in sustaining cell growth of both FH replete and FH depleted cell lines.

To evaluate the roles of Gln and Asn in sustaining energy balance and supporting ATP metabolism needed for growth and survival, we measured the oxygen consumption rate (OCR) and extracellular acidification rate (ECAR) in the presence of Gln, Asn, or both amino acids. Basal respiration and ATP production rates of both cell lines indicated that Gln but not Asn contributes to mitochondrial respiration and maintenance of energy balance (Fig. S1A). Asn had no additive effect on either parameter when added with Gln. UOK262 FH-restored but not FH null cells showed a small decrease in ECAR when supplemented with Gln but not with Asn (Fig. S1B) which implies that Gln contributes to energy generation by fueling the TCA cycle and reducing the burden of glycolysis in the cells with restored functionality of oxidative phosphorylation, as we have previously observed [4].

### Gln and Asn impact the mTORC1 pathway and upregulate UPR gene expression

Gln has been widely reported to be one of the central mediators of the mTORC1 pathway in cancer metabolism [17]. We hypothesized that in the FH deficient cells the activation pattern of main elements of the mTORC1 pathway would be altered when incubating them with Gln, Asn, or both amino acids. To test this hypothesis, we used a phosphoprotein array kit as described in methods to determine the phosphorylation pattern of main downstream proteins of the mTORC1 signaling pathway after 3 h and 48 h of incubation in Gln, Asn, or both. Although no significant changes were observed in the level of phosphorylation of any components of the PI3K-Akt-mTORC1 axis after 3 h of incubation in medium supplemented with either Gln or Asn alone (data not shown), after 48 h, phosphorylation at Ser 235/236 S6 ribosomal protein (S6RP) was increased by both Asn and Gln in both cells (Figure S2A and S2B). These findings strongly suggest that both amino acids—Gln and Asn—are crucial factors that play an important role in the modulation of the mTORC1 complex activity in both cell lines. We performed confirmatory Western blot analysis of downstream elements of mTORC1 pathway in UOK262 cell lines after 48 h of treatment. Both cell lines showed upregulation of pS6-RP after treatment with either Asn or Gln treatment conditions confirming activation of mTORC1 pathway by these nutrients (Figure S2C). Notably, UOK262 cells showed greater increases in both pmTOR and pS6RP levels as compared to UOK262WT cells after stimulation with Asn alone (Fig. S2B, C), which may have been a result of the enhanced Asn auxotrophy of UOK262 cells as compared to their FH-restored counterparts.

For better resolution of the response of UOK262 cells to Gln, Asn, or both nutrients in combination, we performed messenger RNA sequencing of UOK262 FH null and FH-restored cell lines after 96 h incubation with 100 μM Asn, 2 mM Gln, or both combined and performed comprehensive pair-based comparison analysis between cell lines, treatments, and untreated controls. The 96 h time point was chosen for comparison, as it appeared to be the inflection point for growth (Fig. 1). To filter out most differentially expressed genes (DEGs), we set the cut-off criterion of adjusted *p* < 0.05 and |log_2_FC| ≥ 2. Using these criteria, we identified 39 uniquely upregulated genes in UOK262 cells that were treated with both Asn and Gln compared to cells treated only with single amino acid (Table S1). Thirty-five genes were uniquely downregulated under the same conditions. We also identified common genes that were upregulated (11 DEGs) and downregulated (9 DEGs) by Asn and Gln treatments separately but not by combined treatment of UOK262 cells (Table S1).

**Fig. 2:**
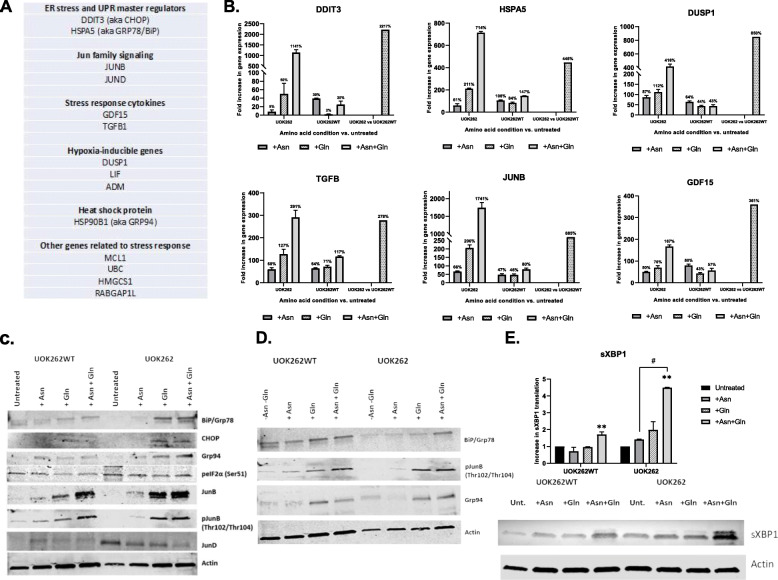
**a** A short list of representative genes relevant to UPR/ER stress-related cellular response identified through enrichment analysis of mRNA-Seq data obtained from UOK262/UOK262WT cell lines incubated with either Asn, Gln, or both for 96 h. **b** UPR/ER stress response related mRNA-Seq gene expression verified by means of RT-PCR. The first two panels of bars at each diagram represent the amino acids treatment resulted in expression fold change compared to untreated control in UOK262 and UOK262WT respectively. The dotted bar on the right side of each diagram represents the fold change of gene expression in UOK262 vs. UOK262WT cells treated with both amino acids (Asn + Gln). **c** Confirmative Western blots of UPR/ER stress-related proteins. The UOK262/UOK262WT cells treated with either Asn, Gln, or both for 96 h before cell homogenization. **d** Western blot analysis of BiP and GRP94 expression and phosphorylation pattern of pJunB proteins in reverse experiment. The UOK262/UOK262WT cells treated with Asn and Gln for 72 h prior to removal either Asn, Gln, or both amino acids for the next 24 h following by cell homogenization. **e** Western blot of a spliced (active) variant of an XBP1 transcription factor in UOK262/UOK262WT after 96 h of incubation with Asn, Gln, or both. Diagram represents quantitative densitometry of the sXBP1 protein under different conditions. For all Western blot experiments 20 μg of total protein loaded at each well, unless stated otherwise. Actin used as a loading control. Statistical analysis was performed to compare the untreated versus treated samples using one-way ANOVA test following by unpaired, two-tailed *t* tests (GraphPad Prism v. 8). (**) *p* < 0.01. The significance between treatment groups shown as (#) *p* < 0.05

Along with universally common signaling pathways implicated in cancer, such as HIF-1alpha, p53, and AP1 pathways, we identified a unique set of genes that were upregulated only in the singular condition of the UOK262 cells incubated in both Gln and Asn and which were not upregulated under Gln or Asn incubation alone (Fig. 2a). These genes were largely related to the unfolded protein response (UPR) or ER stress response pathways, and they were also upregulated in the UOK262 cells relative to UOK262WT cells under the incubation conditions of both Gln and Asn. These mRNA-seq findings were confirmed by RT-PCR studies (Fig. 2b). In the parental line supplied with both amino acids, ASNS transcription increased by an average of 130% compared to the FH+ cells at the same conditions.

Confirmatory Western blot analyses showed a robust increase of expression of UPR regulators BiP, Grp94, CHOP, and JUNB proteins and also an increase in JUNB phosphorylation 96 h after treatment with Gln alone and Gln with Asn in FH null cells (Fig. 2c). To mimic a treatment approach of restricting access of cancer cells to the amino acids, we also performed the reverse experiment at which both cell lines were incubated in medium containing both amino acids for 72 h following removal Asn, Gln, or both amino acids (control) for the next 24 h (Fig. 2d). With both the addition and subtraction studies, the Western blot analysis of phosphorylation state of three key proteins implicated in ER/UPR stress, and the TGFβ pathways, GRP78, pJunB, and GRP94 demonstrated an increased pattern of phosphorylation in the cells exposed to both Asn and Gln compared to the cells without these nutrients. These results suggest possible manipulation of UPR signaling pathways by restricting tumor access to Asn and/or Gln.

To take an orthogonal approach to the induction of UPR in UOK262 and FH+ cells, we examined the ratio of spliced to unspliced XBP1 by qRT-PCR under the same conditions examined in the mRNA-seq analysis. The splicing of XBP1 mRNA by IRE1 upon endoplasmic reticulum stress creates an active isoform of the XBP1 transcription factor (sXBP1), and therefore a change in the ratio of spliced to unspliced XBP1 is an indicator of UPR induction. RT PCR demonstrated that the UOK262 cells showed an increase in sXBP1/uXBP1 ratio after growth in Gln (3.8 ± 0.6 relative to untreated control) and in the combination of Gln plus Asn (6.0 ± 0.7 relative to untreated control) (data not shown). In Fig. 2e, Western blot analysis confirmed a 4.5-fold increase in sXBP1 under the condition of Asn plus Gln in the UOK262 cells relative to Asn or Gln alone. A similar but lesser induction of sXBP1 expression was seen in the UOK262WT cells (1.8-fold) in the presence of both amino acids relative to either amino acid alone. Thus, by both mRNA-seq and examination of sXBP1/uXBP1, there is consistent evidence that UOK262 cells stimulate the UPR when exposed to Asn in the presence of Gln, and less consistent evidence that Asn and Gln together stimulate the UPR in UOK262WT, and that Gln alone stimulates it in UOK262 cells.

We hypothesized that UOK262 cells under ER stress might be more sensitive to other inducers of ER stress in the presence of Asn or relative to UOK262WT cells. However, the Notch signaling inhibitor, FLI06, an autophagy inhibitor, chloroquine, and the GlcNAc phosphotransferase inhibitor tunicamycin did not prove to be significantly more toxic to UOK262 cells in the presence of Asn, and thapsigargin (sarcoplasmic/endoplasmic reticulum Ca^2+^ − ATPase inhibitor) was only modestly more potent in the presence of Asn (Figure S3).

To further understand how Asn affects cellular metabolism in these cell lines, we conducted a stable isotope resolved metabolomic study using ^13^C^15^N-labeled isotopes of Gln and Asn. In these studies, the cells were incubated in unlabeled Gln alone, Asn alone, or Gln and Asn together for 96 h, and then the medium was replaced with the labeled amino acid and the incubation proceeded for another 24 h prior to harvesting. The cells preincubated with Gln and Asn were replaced with ^13^C^15^N labeled Gln/unlabeled Asn or unlabeled Gln/^13^C^15^N labeled Asn to track the contribution of each amino acid to cellular constituents in the presence of both using both NMR and IC-FTMS.

**Fig. 3:**
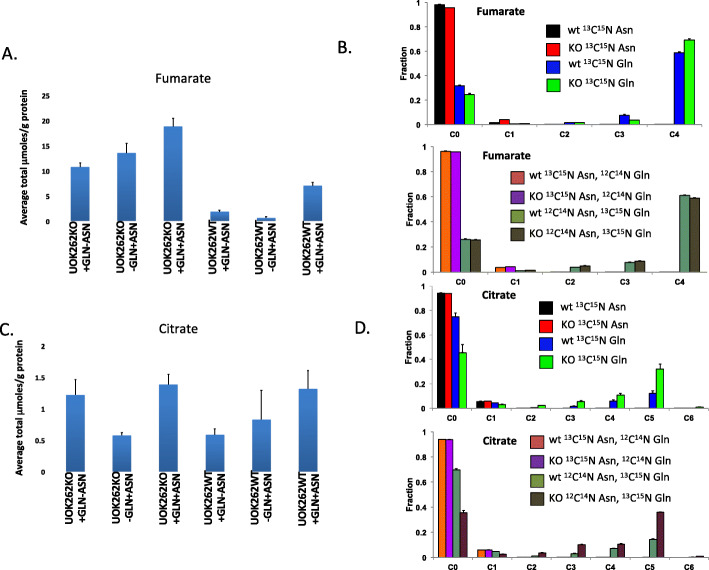
SIRM analysis of fumarate and citrate in UOK262 cells by IC-FTMS under different conditions. Metabolites were resolved and quantified as described in the “Methods” section, and the absolute concentrations and fraction enrichments are given for both UOK262WT (WT) and UOK262FH−/− (KO) under different treatment conditions. Means and standard errors are shown. An unpaired two-tailed *t* test was used for statistical comparisons. Cx_Ny are isotopologues that contain x^13^C and y^15^N atoms. **a** Absolute amounts of total fumarate in μmole/g. UOK262 *p* = 0.0158 for +Gln + Asn versus +Gln-Asn; *p* = 0.102 for +Gln + Asn versus -Gln + Asn: WT *p* = 0.0044 for +Gln + Asn versus +Gln-Asn; *p* = 0.033 for -Gln + Asn versus +Gln + Asn. **b** Fractional ^13^C enrichment in fumarate isotopologues. Upper: WT versus KO for ^13^C^15^N Asn alone and for ^13^C^15^N Gln alone.  WT ^13^C^15^N Asn,  KO ^13^C^15^N Asn,  WT ^13^C^15^N Gln,  KO ^13^C^15^N Gln. Lower: WT versus KO for ^13^C^15^N Asn with ^12^C^14^N (unlabeled) Gln and for ^12^C^14^N (unlabeled) Asn + ^13^C^15^N Gln.  WT ^13^C^15^N Asn plus ^12^C^14^N Gln,  KO ^13^C^15^N Asn plus ^12^C^14^N Gln,  WT ^12^C^14^N Asn plus ^13^C^15^N Gln,  KO ^12^C^14^N Asn plus ^13^C^15^N Gln. **c** Absolute amounts of total citrate in μmole/g. UOK262 *p* = 0.607 for +Q + N versus +Q-N; *p* = 0.0083 for +-Q + N versus +Q + N: WT *p* = 0.0723 for +Q + N versus +Q-N; *p* = 0.452 for +-Q + N versus +Q + N. **d** Fractional ^13^C enrichment in citrate. WT Gln versus KO Gln *p* = 0.0082; WT Gln + Asn vs KO Gln + Asn *p* = 0.0002. Upper: WT versus KO for ^13^C^15^N Asn alone and for ^13^C^15^N Gln alone.  WT ^13^C^15^N Asn,  KO ^13^C^15^N Asn,  WT ^13^C^15^N Gln,  KO ^13^C^15^N Gln. Lower: WT versus KO for ^13^C^15^N Asn with ^12^C^14^N (unlabeled) Gln and for ^12^C^14^N (unlabeled) Asn + ^13^C^15^N Gln.  WT ^13^C^15^N Asn plus ^12^C^14^N Gln,  KO ^13^C^15^N Asn plus ^12^C^14^N Gln,  WT ^12^C^14^N Asn plus ^13^C^15^N Gln,  KO ^12^C^14^N Asn plus ^13^C^15^N Gln

Fumarate served as an important internal control, with the expectation that fumarate levels would be higher in the UOK262 cells than in the UOK262WT cells. This is the case, as can be seen in Fig. 3a, and by HSQC (Fig. S4), which demonstrates the total fumarate levels in the two cell lines under the three conditions (where labeled Gln/unlabeled Asn and unlabeled Gln/labeled Asn are grouped together). As can be seen in Fig. 3b, all of the labeling of fumarate comes from the labeled Gln and none comes from the labeled Asn. In contrast, there are no significant differences in intracellular citrate pools between the two cell lines, although once again all of the label comes from Gln, not Asn.

**Fig. 4:**
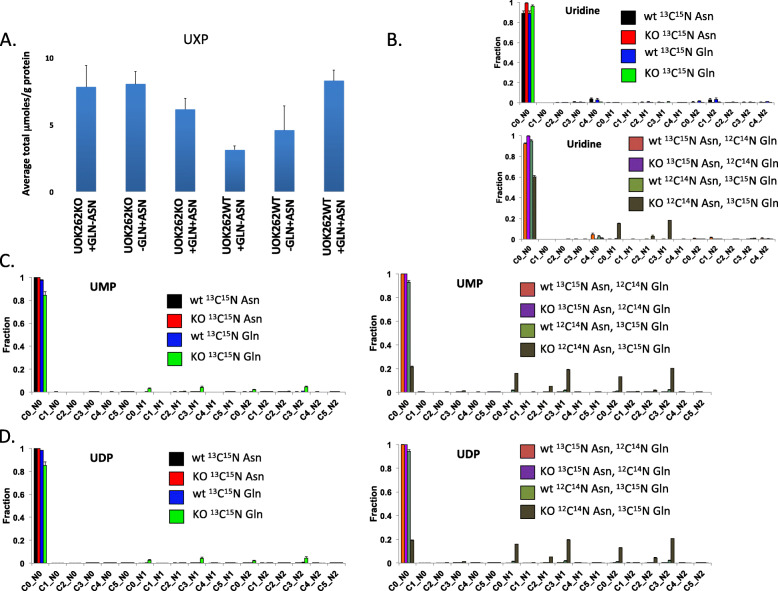
^13^C enrichments from Asp and Gln in uridine nucleotides. Nucleotides for UOK262WT (WT) and UOK252FH−/− (KO) under different conditions were quantified by IC-MS as described in the “Methods” section. An unpaired two-tailed *t* test was used for statistical comparisons. **a** Total uridine in μmole/g. UOK262 + Gln + Asn versus +Gln-Asn *p* = 0.148; + Gln + Asn versus –Gln + Asn *p* = 0.422. **b**, **c**, **d**^13^C fractional enrichment from ^13^C/^15^N Asn and ^13^C/^15^N glutamine in uridine, uridine monophosphate, and uridine diphosphate, respectively. Cx_Ny are isotopologues that contain x^13^C and y^15^N atoms. Upper: WT versus KO for ^13^C^15^N Asn alone and for ^13^C^15^N Gln alone.  WT ^13^C^15^N Asn,  KO ^13^C^15^N Asn,  WT ^13^C^15^N Gln,  KO ^13^C^15^N Gln. Lower: WT versus KO for ^13^C^15^N Asn with ^12^C^14^N (unlabeled) Gln and for ^12^C^14^N (unlabeled) Asn + ^13^C^15^N Gln.  WT ^13^C^15^N Asn plus ^12^C^14^N Gln,  KO ^13^C^15^N Asn plus ^12^C^14^N Gln,  WT ^12^C^14^N Asn plus ^13^C^15^N Gln,  KO ^12^C^14^N Asn plus ^13^C^15^N Gln

The de novo synthesis of uracil from labeled Gln into uridine nucleotides was strikingly stimulated by Asn in the presence of Gln in the UOK262 cells. Figure 4a shows a slight increase in the total uridine nucleotide pool (UXP) in the UOK262WT cells in the presence of Gln and Asn compared to either amino acid alone, and Fig. 4b shows a significant fraction of uridine becomes labeled only in the UOK262 cells in the Asn plus Gln condition, with all of the label coming from Gln. This becomes more apparent as UXP undergoes metabolic transformation, with very striking incorporation of label into UMP (Fig. 4c) and UDP (Fig. 4d) only in UOK262 cells under the condition of Asn plus Gln, and again with all of the label coming from Gln.

**Fig. 5:**
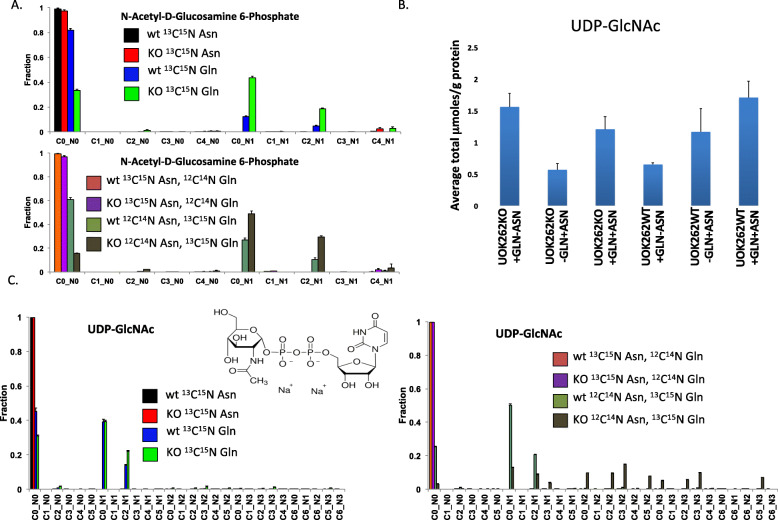
^13^C enrichments from labeled Asp or Gln in UDPGlcNAc and metabolic subunits. N-Acetyl-D-Glucosamine 6-Phosphate and UDPGlcNAc were quantified for UOK262WT (WT) and UOK252FH−/− (KO) under different conditions by IC-MS as described in the “Methods” section. **a** Fractional enrichment in isotopologues of N-Acetyl-D-Glucosamine 6-Phosphate. **b** Total UDPGlcNAc in μmol/g protein. UOK262 + Q + N versus UOK262 + Q-N *p* = 0.0601; 262WT + Q + N versus +Q-N *p* = 0.0164, +Q + N versus –Q + N *p* = 0.296. **c** Fractional enrichment in isotopologues of UDPGlcNAc. Cx_Ny are isotopologues that contain x^13^C and y^15^N atoms. Upper (Panel A) and Left (Panel C): WT versus KO for ^13^C^15^N Asn alone and for ^13^C^15^N Gln alone.  WT ^13^C^15^N Asn,  KO ^13^C^15^N Asn,  WT ^13^C^15^N Gln,  KO ^13^C^15^N Gln. Lower (Panel A) and right (Panel C): WT versus KO for ^13^C^15^N Asn with ^12^C^14^N (unlabeled) Gln and for ^12^C^14^N (unlabeled) Asn + ^13^C^15^N Gln.  WT ^13^C^15^N Asn plus ^12^C^14^N Gln,  KO ^13^C^15^N Asn plus ^12^C^14^N Gln,  WT ^12^C^14^N Asn plus ^13^C^15^N Gln,  KO ^12^C^14^N Asn plus ^13^C^15^N Gln

The other pathway showing significant label incorporation in the UOK262 cells was the synthesis of N-acetyl-D-glucosamine phosphate (GlcNAc) (Fig. 5a), where both Gln and Gln plus Asn stimulate the incorporation of Gln-derived label into GlcNAc and UDP-GlcNAc. As can be seen in Fig. 5b, there are no significant differences in total UDP-GlcNAc pools under the different conditions. However, as can be seen in Fig. 5c, Gln labels a significant percentage of the UDP-GlcNAc pool, and there was a much greater degree of labeling when the cells were co-incubated with Asn. Almost all of the UDP-GlcNAc is labeled in the UOK262 cells in the presence of both Asn and Gln, suggesting active synthesis and subsequent metabolism of UDP-GlcNAc in this cell line under this condition. The C2 label is likely from the acetyl group on GlcNAc, derived from labeled Gln that underwent reductive carboxylation to form citrate, which then undergoes cleavage in the cytosol to form acetyl-CoA, in both UOK262 and UOK262WT in the presence of Gln with or without Asn. The C3 label of UDP-GlcNAc likely derived from de novo biosynthesis of uridine from Gln. This occurred only in cells UOK262 cells grown in the presence of Gln plus Asn. The M + 3 aspartate was also derived from the reductive carboxylation of Gln [4]. Similarly, N-acetyl galactosamine (Fig. 5d) and UDP-GalNAc (Fig. 5e) are labeled by Gln but not by Asn, but with more Gln labeling in the presence of Asn. For both UDP-GlcNAc and UDP-GalNAc pools, labeling is nearly complete for the UOK262 cells in the presence of Asn and Gln, even though all of the label is coming from Gln. This implies that Asn is not substrate for the amido transferases, but this activity is stimulated by Asn.

**Fig. 6:**
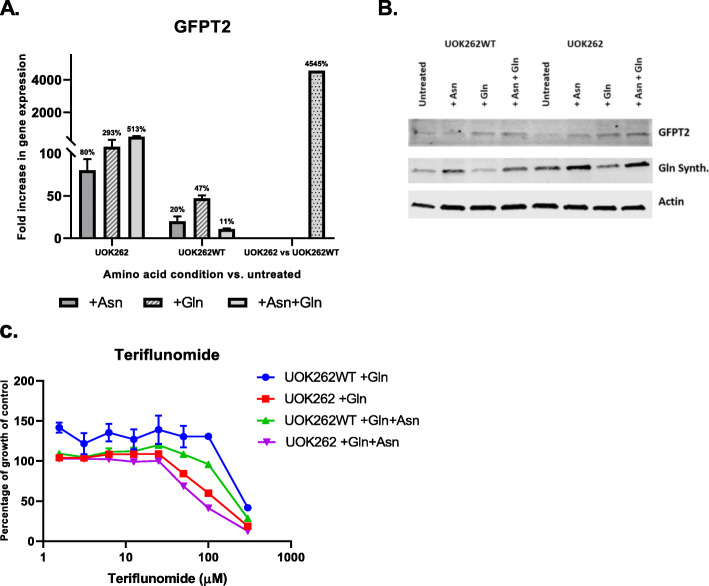
**a** RT-PCR results for GFPT2 gene. The first two panels of bars at each diagram represent the amino acids treatment resulted in expression fold change compared to untreated control in UOK262 (KO) and UOK262WT respectively. The dotted bar on the right side of each diagram represents the fold change of gene expression in UOK262 vs. UOK262WT cells treated with both amino acids (Asn + Gln). **b** Confirmative Western blot analysis of GFPT2 and Gln synthase proteins. The UOK262/UOK262WT cells treated with either Asn, Gln, or both for 96 h before cell homogenization. Twenty microgram of total protein loaded at each well unless stated otherwise. Actin used as a loading control. **c** Cytotoxicity effect of a pyrimidine inhibitor, teriflunomide, on UOK262/UOK262WT cell lines. The curves represent the percentage of untreated control and calculated after 96 h of incubation of the cells in different concentration of teriflunomide in the presence of either Asn, Gln, or both

Previous reports have linked sXBP1 to the induction of Gln fructose-6-phosphate amidotransferase 1 (GFAT1), the murine homolog of the human GFPT2 gene, which is the rate limiting step in the production of UDP-GlcNac [18]. We therefore sought to determine whether there was evidence that induction of these two genes in the UOK262 cells in the presence of Asn and Gln provided a link between the RNAseq data showing the induction of UPR and the metabolomic data showing the increased synthesis of uridine metabolites and glycans. As shown in Fig. 6, GFPT2 RNA (Fig. 6a) and protein (Fig. 6b) in increased in UOK262 cells in the presence of Gln and Asn. The protein expression of Gln synthetase, which has been reported to be stimulated by Asn in T47D breast cancer cells [19] appeared to be upregulated in both UOK262 and UOK262WT cells in the presence of Asn, and did not appear to depend on the presence of Gln in these cells.

Finally, although the metabolic data show strong evidence that the presence of Asn alters uridine metabolism, it shows scant evidence that Asn is actually catabolized into any of these cellular constituents. The remaining possibilities for the fate of Asn are that asparagine is incorporated into proteins without any metabolic transformation, or that it is used as an exchange factor for the uptake of other amino acids from the medium [20].

## Discussion

Three intriguing findings were observed after the exposure of FH-deficient UOK262 and the FH-restored UOK262WT cells to Asn in Gln-containing medium. First, mRNA-seq studies showed that the UPR response was induced in the UOK262 FH-deficient cells after exposure to Asn plus Gln, but not after exposure to Gln or Asn alone. Second, stable isotope-resolved metabolomics showed that the same cells under the same nutrient condition of Asn plus Gln increased the de novo synthesis of uridine phosphates and amino sugars of the hexosamine biosynthetic pathway (HBP), but not after exposure to Gln or Asn alone. Third, FH repletion of the UOK262 cells abrogated the effect of Asn in regard to both UPR and HBP induction.

In this study, Asn and Gln, together but not alone, increased the expression of GFPT2. These findings were consistent with a demonstrated ability of the amino acids to shift the XBP1 mRNA balance to its spliced, isoform when added to parental cell line in conjunction, suggesting that HBP is mediated through activation of XBP1 by the two amino acids. XBP1 mRNA undergoes splicing to become an active transcription factor triggering of unfolded protein response cascade in response to cellular stress inflicted by the accumulation of abnormal proteins. The spliced XBP1 transcription factor has been broadly reported as a key element of regulation of gene expression and has been shown to be implicated in multiple pathological conditions including diabetes, Alzheimer’s disease and various types of cancer [21–25]. The role of the XBP1 in the context of human malignancies has been broadly investigated and reported to have a pivotal role in cell survival and proliferation. Recent studies found that XBP1 depletion from triple negative breast cancer cell models inhibited tumor growth and tumor relapse, and the active form of XBP1 was linked to the activation of HIF1α pathway and angiogenesis, and associated with poor prognosis [26]. However, the role of XBP1 in the progression of FH mutated renal cell carcinoma has been unrecognized. In this study, we demonstrate that activation of XBP1 and the UPR response of increased GFPT2 can occur as a result of nutrient conditions of the combination of two amino acids—Asn and Gln.

Our findings demonstrate that UOK262 cells in the presence of both Asn and Gln use the hexosamine biosynthetic pathway to produce UDP-GlcNAc and UDP-GalNAc, which are respectively the substrates for N-glycosylation and O-glycosylation post-translational modifications of proteins. Both Wang et al. [27] and Denzel et al. [28] have reported that sXBP1, which is spliced to its active form during ER stress, activates hexosamine pathway synthesis. Wang et al. showed sXBP1-induction of murine GFAT1, which encodes Gln-fructose-6-phosphate amidotransferase (GFPT), increased the synthesis of GlcNAc in cardiomyocytes, which provided cardioprotection during ischemia/reperfusion stress. Denzel et al. created ER stress in *C. elegans* by exposure to tunicamycin, isolated tunicamycin-resistant strains, and found gain-of-function point mutations of the Gln-fructose 6-phosphate gene, GFAT1, leading to elevated UDP-HexNAc levels. *C. elegans* that have been engineered to express these novel gain of function mutations showed a pro-survival phenotype, with extended life spans observed.

Our studies also show an analogous induction of UPR and HBP, but in the surprising context of Asn exposure of cancer cells lacking FH activity. While the protective role of N- and O-glycosylation against ER stress has been well documented in several experimental systems, the exact mechanism(s) involved are not known [18]. It is not known, for example, if these protein modifications affect critical individual proteins, or whether they aid either in the recovery or in the enhanced clearance of misfolded proteins [18].

At present, the stimulus for activation of UPR and ER stress responses in UOK262 cells grown in the presence of Gln and Asn is not known. Fumarate-mediated S-succination of protein thiol residues was previously associated with ER stress and UPR activation in differentiating adipocytes under high glucose conditions [29, 30]. Increased protein S-succination is a well-characterized consequence of elevated fumarate in FH^−/−^ tumors and cells, and prevalent protein S-succination has observed both histologically and biochemically in tumors derived from HLRCC patients [31, 32]. In the present study, we found that growth of UOK262 cells with Gln and Asn resulted in a nearly 2-fold increase in intracellular fumarate levels. We propose that the increased intracellular fumarate in UOK262 cells stimulated by Gln and Asn in our study led to increased proteostatic stress due to increased protein S-succination, which led to stimulation of the UPR and ER stress responses.

It is of interest that Asn did not enhance the effect of tunicamycin in UOK262 cells. Since tunicamycin inhibits N-glycosylation, we expected that it would have a selectively deleterious effect on these cells in the presence of Asn. This observation raises the possibility that O-glycosylation may be more important than N-glycosylation for the pro-survival effect of the activation of the HBP in these cells. Cellular proteins can be modified by O-glycosylation on the hydroxyl groups of threonine and serine residues and these modifications have been linked to improved cellular survival in cardiomyocytes [33] as well as different cancers [34].

Our observation of the elevated activation of XBP1 protein and its reported importance as a pivotal mediator of both processes, UPR and HBP, raises the possibility of exploring the XBP1 as a potential therapeutic target. Recent studies demonstrated the anticancer ability of specific inhibitors of IRE1α-XBP1 axis in vitro and in vivo. Acridine derivatives, for example, recently reported to show cytotoxicity effect on multiple myeloma cell lines, whereas a novel chemical-STF-083010 has been demonstrated to reverse tamoxifen-related drug resistance in breast cancer [35, 36]. Therefore, these and additional compounds could be utilized to test their potential anti-cancer ability in the treatment of type 2 papillary renal cell carcinoma.

Although we have identified pathways that are activated by Asn + Gln, further studies will be required to determine whether disruption of HBP can prevent the effect of ASN on proliferation, and whether a similar relation of Asn exposure to HBP induction can be observed in other cell lines. In addition, whether the effect of Asn in the presence of Gln on FH-deficient UOK262 cells is due to the overall disruption of central metabolism due to the genetic defect or caused directly by the generation excess fumarate and subsequent protein modification, remains to be determined.

## Conclusions

In this report we show by RNAseq that addition of the amino acid asparagine to cell culture medium containing glutamine triggers the UPR in an FH-deficient renal cell carcinoma cell line but not in the FH-repleted control cell line. Further, by using stable isotope resolved metabolomics, we show that the UPR response was associated with increase in glutamine—but not asparagine—labeling of uridine phosphates and hexosamine metabolites indicative of the activation of the HBP, observed again in the FH-deficient cell line but not in the FH-replete cell line. We show evidence that the link between UPR and HBP may be due to UPR-induced spliced XBP1 induction of the expression of the HBP regulator GFPT2. Therefore, our observations show unexpected metabolic effects resulting from changing amino acid nutrient conditions by the addition of asparagine that is dependent upon the underlying metabolic dysregulation in the cells. Since therapeutic agents are available that can deplete systemic asparagine, this work may have potential therapeutic implications.

## Supplementary information

**Additional file 1: Figure S1**. Quantitation of Basal Respiration (A), ATP production (B), Glycolysis (E) and Glycolytic Reserve (F) of UOK262/UOK262WT cells under different treatment conditions as measured by XF96 Extracellular Flux Analyzer (C-D and G-H)). 24 x 10^3^ cells/well were seeded 24 h prior to the beginning of the experiment. Basal levels of OCR and proton production were first quantified over approximately 20–25 minutes followed by addition specific inhibitors according to the manufacturer protocol. Three to five independent experimental repeats were carried out. Statistical analysis was performed to compare the untreated versus treated samples using one-way ANOVA test following by unpaired, two-tailed t-tests (GraphPad Prism v. 8). (*)P < 0.05; (**)P < 0.01; (***)P < 0.0001. The significance between treatment groups shown as (#)P < 0.05. **Figure S2.** (A**)** Representative 16-pan slide of PathScan Akt Signaling Antibody Array kit performed on UOK262/UOK262WT cells at 3 h and 48 h of incubation in the presence of 100 μM Asn, 2 mM Gln, both amino acids and untreated controls. Fluorescent readout acquisition obtained using the Odyssey Imaging System. Each horizontal pair of dots represents a specific phosphorylated element of the Akt pathway. Three independent experimental repeats were carried out. (B) Change in phosphorylation pattern of pS6RP after different treatments of UOK62/UOK262WT cells after 3 h (top) and 48 h (bottom) of incubation obtained through PathScan antibody array kit. Quantitation performed using ImageJ software. (C) Western Blot analysis of phosphorylation pattern of mTOR, S6 kinase, S6 ribosomal protein, and 4E-BP1 proteins after 48 h of treatments. The UOK262/UOK262WT cells treated with 100 μM Asn, 2 mM Gln or both for 48 h along with untreated control following by cell homogenization. For all Western Blot experiments 20 μg of total protein loaded at each well, unless stated otherwise. Actin used as a loading control. Statistical analysis was performed to compare the untreated versus treated samples using one-way ANOVA test following by unpaired, two-tailed t-tests (GraphPad Prism v. 8). (*)P < 0.05; (**)P < 0.01; (***)P < 0.0001. The significance between treatment groups shown as (#)P < 0.05. **Figure S3**. Cytotoxicity curves for a Notch signaling inhibitor Fli06 (A), an autophagy inhibitor chloroquine (B), an UPR stress inducer tunicamycin (C) and a specific inhibitor of the sarcoplasmic/endoplasmic reticulum Ca^2+^-ATPase (SERCA), thapsigargin (D) as measured on UOK262/UOK262WT cells under different treatment conditions after 96 h of incubation. The curves represent the percentage of the untreated control. Five independent experimental repeats were carried out. **Figure S4**. HSQC analysis of UOK262 cells treated with ^13^C^15^N Gln + ^12^C^14^N Asn. Spectra were recorded as described in the methods. Top KO cells, bottom wt cells. The ^1^H{^13^C} HSQC spectrum selects protons attached directly to ^13^C only. Gln is taken up by the cells and converted to Glu, GSH and fumarate. The free induction decays were multiplied by a 4-Hz line broadening exponential prior to fourier transformation. **Table S1.** List of treatment unique genes produced by comparison analysis of mRNA-Seq data. Four groups of genes generated based on the gene expression pattern (upregulated and downregulated) affected by incubation with either Asn and Gln or both amino acids.

## Data Availability

The datasets collected during and/or analyzed during the current study are available from the corresponding author on reasonable request.

## Abbreviations

HLRCC: Hereditary leiomyomatosis and renal cell carcinoma
FH: Fumarate hydratase
PPP: Pentose phosphate pathway
HIF: Hypoxia inducible factor
VHL: Von Hippel Lindau
TCA: Tricarboxylic acid
ASNS: Asparagine synthetase
ER: Endoplasmic reticulum
SRB: Sulforhodamine B
UPR: Unfolded protein response
S6RP: S6 ribosomal protein
SIRM: Stable isotope resolved metabolomics
OCR: Oxygen consumption rate
ECAR: Extracellular acidification rate
GFPT: Gln-fructose-6-phosphate amidotransferase
