# Supplementary material for: Fumarate hydratase-deficient renal cell carcinoma cells respond to asparagine by activation of the unfolded protein response and stimulation of the hexosamine biosynthetic pathway
Source: Cancer Metab. 2020 Aug 3;8:7. doi: 10.1186/s40170-020-00214-9 (PMC7397616; doi:10.1186/s40170-020-00214-9)
Supplement: Supplementary file 1 — Additional file 1: Figure S1. Quantitation of Basal Respiration (A), ATP production (B), Glycolysis (E) and Glycolytic Reserve (F) of UOK262/UOK262WT cells under different treatment conditions as measured by XF96 Extracellular Flux Analyzer (C-D and G-H)). 24 x 103 cells/well were seeded 24 h prior to the beginning of the experiment. Basal levels of OCR and proton production were first quantified over approximately 20–25 minutes followed by addition specific inhibitors according to the manufacturer protocol. Three to five independent experimental repeats were carried out. Statistical analysis was performed to compare the untreated versus treated samples using one-way ANOVA test following by unpaired, two-tailed t-tests (GraphPad Prism v. 8). (*)P < 0.05; (**)P < 0.01; (***)P < 0.0001. The significance between treatment groups shown as (#)P < 0.05. Figure S2. (A) Representative 16-pan slide of PathScan Akt Signaling Antibody Array kit performed on UOK262/UOK262WT cells at 3 h and 48 h of incubation in the presence of 100 μM Asn, 2 mM Gln, both amino acids and untreated controls. Fluorescent readout acquisition obtained using the Odyssey Imaging System. Each horizontal pair of dots represents a specific phosphorylated element of the Akt pathway. Three independent experimental repeats were carried out. (B) Change in phosphorylation pattern of pS6RP after different treatments of UOK62/UOK262WT cells after 3 h (top) and 48 h (bottom) of incubation obtained through PathScan antibody array kit. Quantitation performed using ImageJ software. (C) Western Blot analysis of phosphorylation pattern of mTOR, S6 kinase, S6 ribosomal protein, and 4E-BP1 proteins after 48 h of treatments. The UOK262/UOK262WT cells treated with 100 μM Asn, 2 mM Gln or both for 48 h along with untreated control following by cell homogenization. For all Western Blot experiments 20 μg of total protein loaded at each well, unless stated otherwise. Actin used as a loading control. Statistical analysis was [file 40170_2020_214_MOESM1_ESM.pdf]

Figure S1

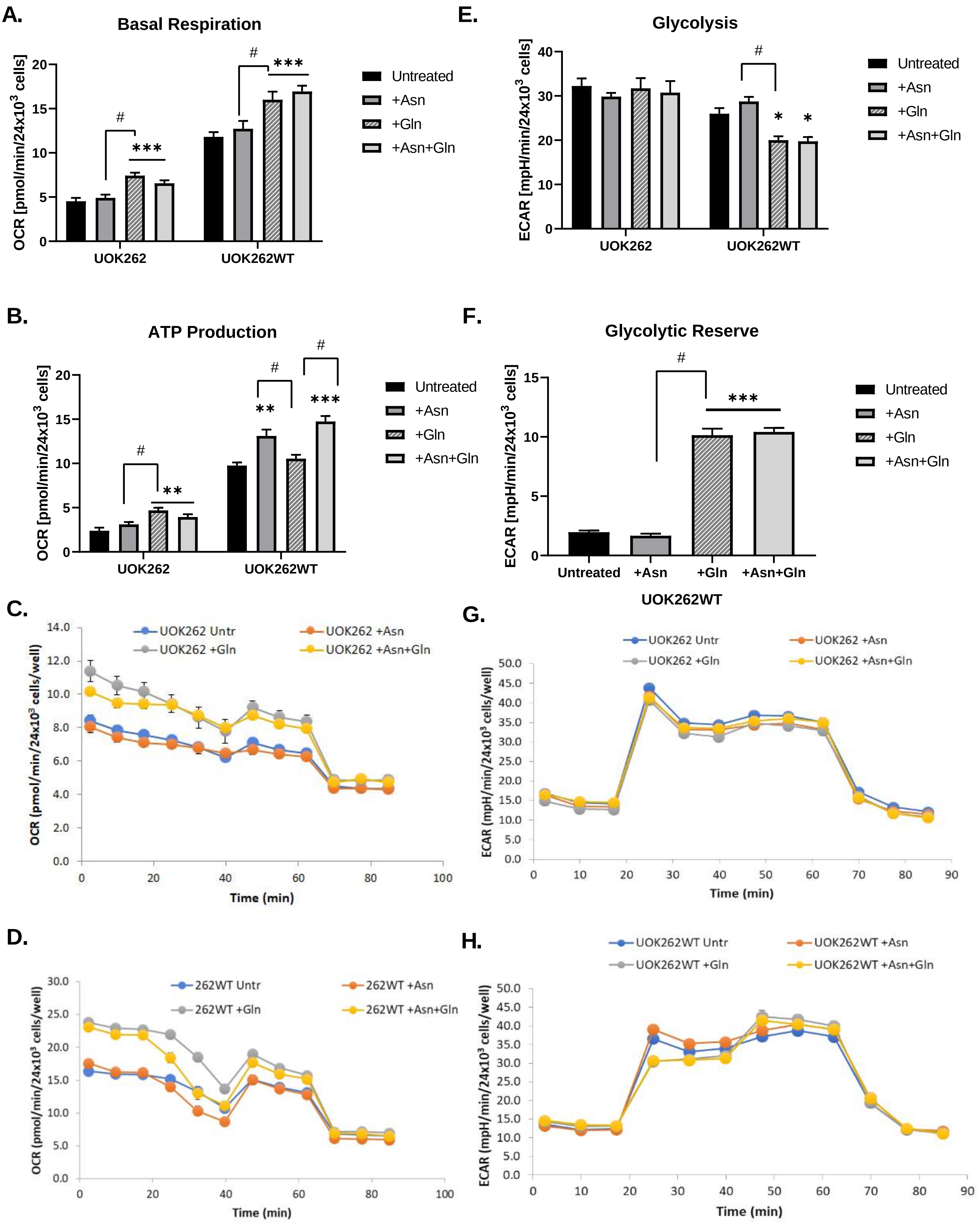

A.
Basal Respiration
OCR [pmol/min/24x10³ cells]
UOK262
UOK262WT
Untreated
+Asn
+Gln
+Asn+Gln
B.
ATP Production
OCR [pmol/min/24x10³ cells]
UOK262
UOK262WT
Untreated
+Asn
+Gln
+Asn+Gln
C.
UOK262 Untr
UOK262 +Asn
UOK262 +Gln
UOK262 +Asn+Gln
OCR (pmol/min/24x10³ cells/well)
Time (min)
D.
262WT Untr
262WT +Asn
262WT +Gln
262WT +Asn+Gln
OCR (pmol/min/24x10³ cells/well)
Time (min)
E.
Glycolysis
ECAR [mpH/min/24x10³ cells]
UOK262
UOK262WT
Untreated
+Asn
+Gln
+Asn+Gln
F.
Glycolytic Reserve
ECAR [mpH/min/24x10³ cells]
Untreated
+Asn
+Gln
+Asn+Gln
UOK262WT
G.
UOK262 Untr
UOK262 +Asn
UOK262 +Gln
UOK262 +Asn+Gln
ECAR (mpH/min/24x10³ cells/well)
Time (min)
H.
UOK262WT Untr
UOK262WT +Asn
UOK262WT +Gln
UOK262WT +Asn+Gln
ECAR (mpH/min/24x10³ cells/well)
Time (min)

Figure S2

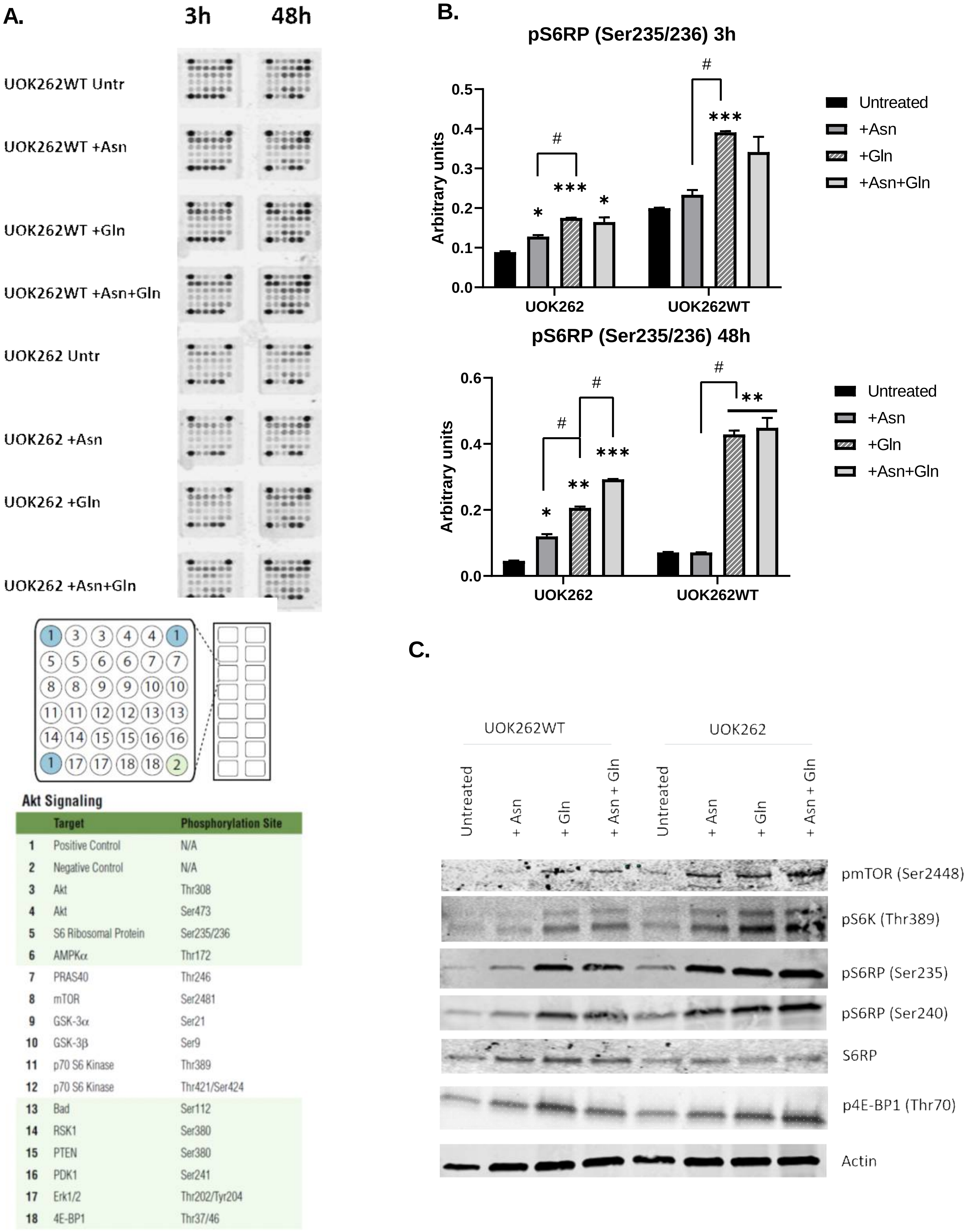
A.
3h
48h
UOK262WT Untr
UOK262WT +Asn
UOK262WT +Gln
UOK262WT +Asn+Gln
UOK262 Untr
UOK262 +Asn
UOK262 +Gln
UOK262 +Asn+Gln
Akt Signaling
Target | Phosphorylation Site
1 Positive Control N/A
2 Negative Control N/A
3 Akt Thr308
4 Akt Ser473
5 S6 Ribosomal Protein Ser235/236
6 AMPKα Thr172
7 PRAS40 Thr246
8 mTOR Ser2481
9 GSK-3α Ser21
10 GSK-3β Ser9
11 p70 S6 Kinase Thr389
12 p70 S6 Kinase Thr421/Ser424
13 Bad Ser112
14 RSK1 Ser380
15 PTEN Ser380
16 PDK1 Ser241
17 Erk1/2 Thr202/Tyr204
18 4E-BP1 Thr37/46
B.
pS6RP (Ser235/236) 3h
Arbitrary units
UOK262
UOK262WT
Untreated
+Asn
+Gln
+Asn+Gln
pS6RP (Ser235/236) 48h
C.
UOK262WT
UOK262
Untreated
+ Asn
+ Gln
+ Asn + Gln
pmTOR (Ser2448)
pS6K (Thr389)
pS6RP (Ser235)
pS6RP (Ser240)
S6RP
p4E-BP1 (Thr70)
Actin

Figure S3

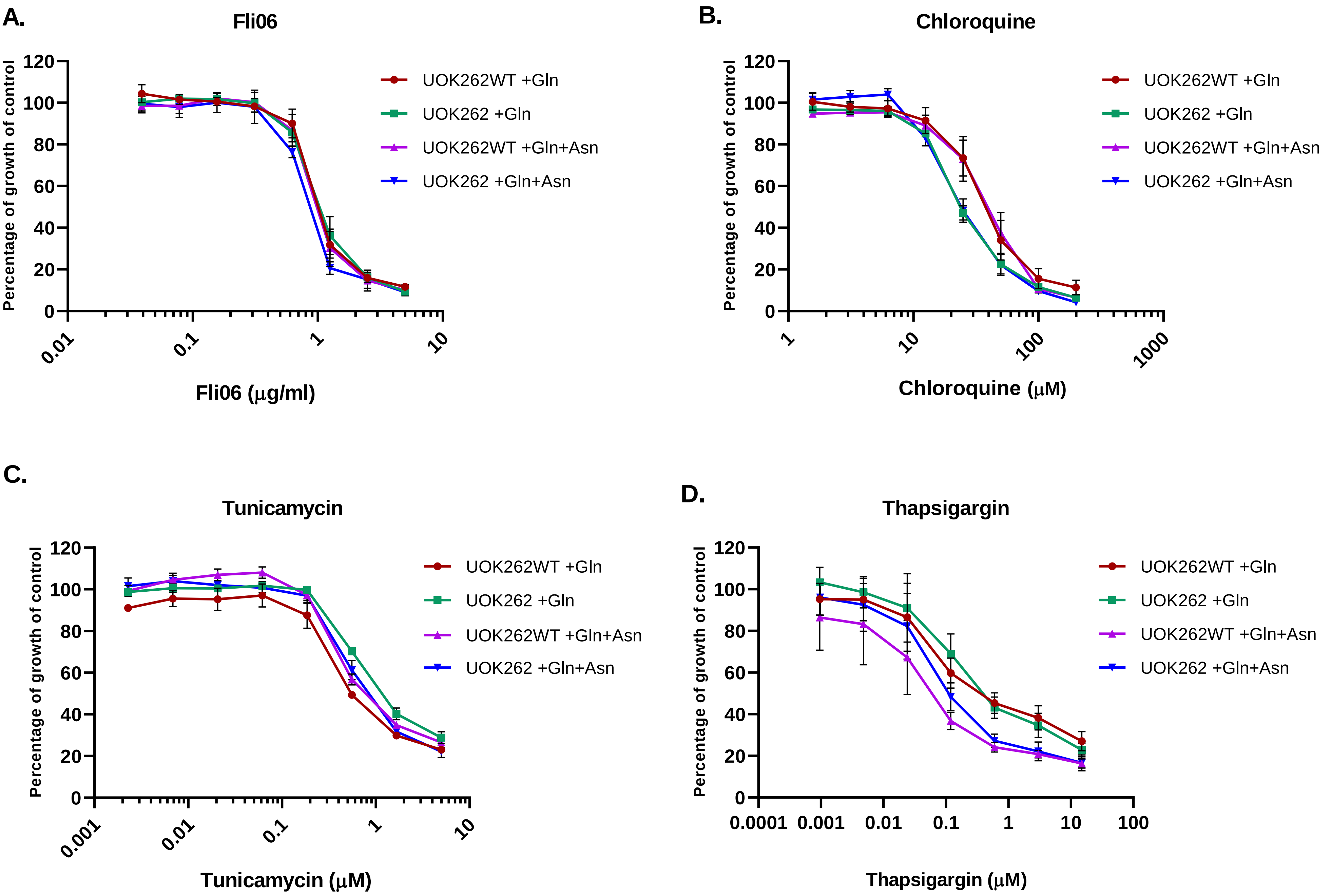

A.
Fli06
Percentage of growth of control
0
20
40
60
80
100
120
0.01
0.1
1
10
Fli06 (μg/ml)
UOK262WT +Gln
UOK262 +Gln
UOK262WT +Gln+Asn
UOK262 +Gln+Asn
B.
Chloroquine
Percentage of growth of control
0
20
40
60
80
100
120
1
10
100
1000
Chloroquine (μM)
UOK262WT +Gln
UOK262 +Gln
UOK262WT +Gln+Asn
UOK262 +Gln+Asn
C.
Tunicamycin
Percentage of growth of control
0
20
40
60
80
100
120
0.001
0.01
0.1
1
10
Tunicamycin (μM)
UOK262WT +Gln
UOK262 +Gln
UOK262WT +Gln+Asn
UOK262 +Gln+Asn
D.
Thapsigargin
Percentage of growth of control
0
20
40
60
80
100
120
0.0001
0.001
0.01
0.1
1
10
100
Thapsigargin (μM)
UOK262WT +Gln
UOK262 +Gln
UOK262WT +Gln+Asn
UOK262 +Gln+Asn

Figure S4

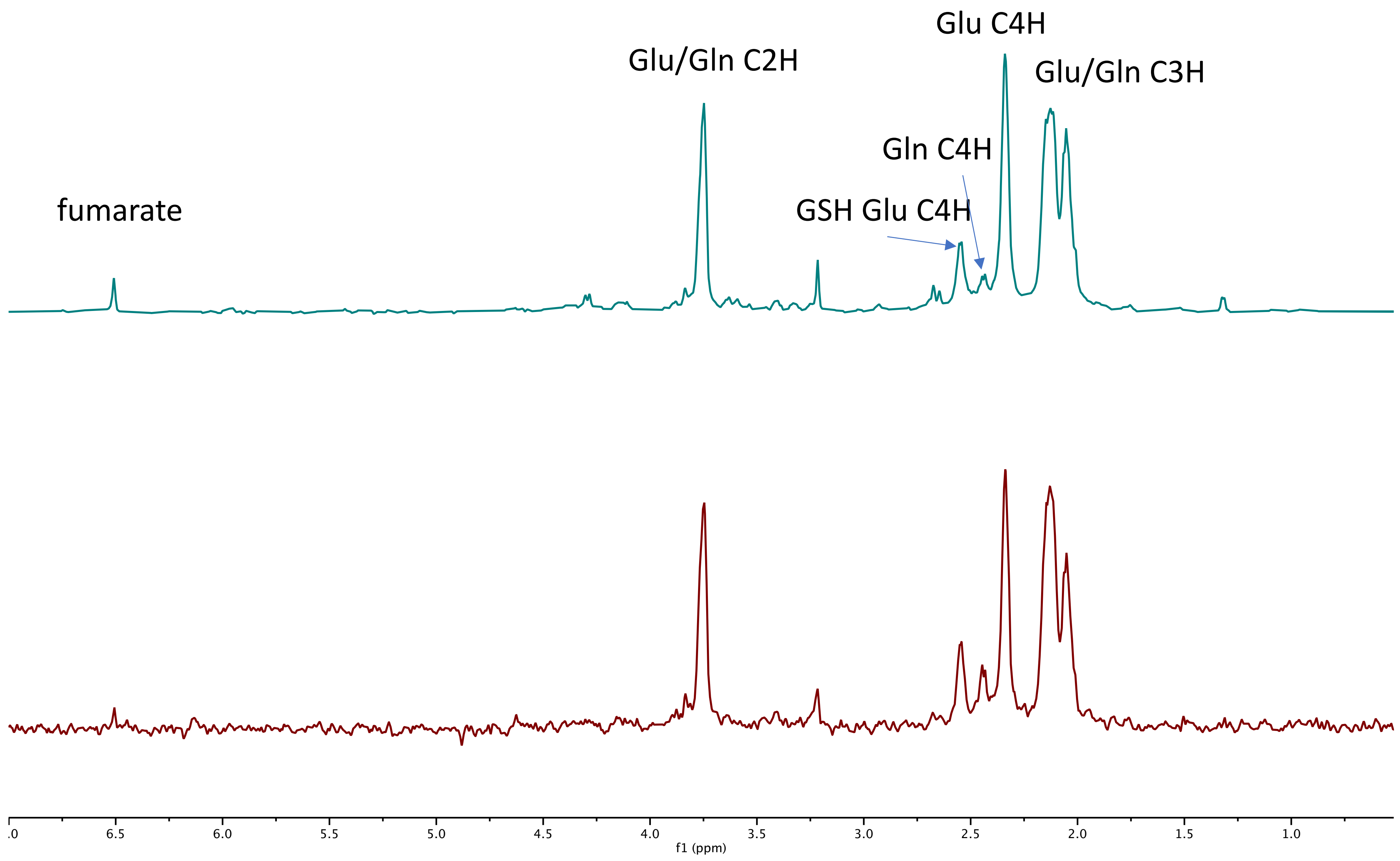

fumarate
Glu/Gln C2H
GSH Glu C4H
Gln C4H
Glu C4H
Glu/Gln C3H
.0
6.5
6.0
5.5
5.0
4.5
4.0
3.5
3.0
2.5
2.0
1.5
1.0
f1 (ppm)

Table S1

**Upregulated by Asn+Gln but not Asn or Gln**	HSP90B1 GDF15 ZFP36L1 NDRG1 SLC20A1 SLC2A1 PHLDA1 GPRC5A LIMA1 BHLHE40 MCL1 DUSP1 PPP1R15 CSNK1E SAT1 HERPUD1 TNFSF9 TGFB1 HMGCS1 ACSL4 TM4S1 FDFT1 LIF JUND PPP1R15B DNAJB1 RABGAP1L UBC BTG1 HIST1H1C JUNB GABA RAPL1 ADM COLEC10 F3 HSPA5 DDIT3 KLF6
**Downregulated by Asn+Gln but not Asn or Gln**	MT-ND2 TAGLN NDST1 EIF3A MDM2 FUS MAT2A SFPQ MICALL1 NPLOC4 ABCA3 HNRNPM SPRR2A SUPT16H SMARCC1 NOP56 CAD MEGF8 ANKRD52 CBX6 SLC35A4 TAF15 SCRIB SH3BP5L MAPKAP1 STARD7 C15orf52 SETD7 IFI16 RCAN1 OBSCN CUL3 MT-ND1 CSK EIF5
**Upregulated by Asn and Gln but not Asn+Gln**	SAA2 STAT1 PDZK1IP1 FSTL1 GSN ANXA4 TMEM2 DHCR24 IDH2 HAVCR1 SAA1
**Downregulated by Asn and Gln but not Asn+Gln**	WARS PSAT1 PPP1R15A ARHGEF2 ASNS HIST1H2AC STC2 UPP1 G0S2
